# An Efficient Multi-Dimensional Resource Allocation Mechanism for Beam-Hopping in LEO Satellite Network

**DOI:** 10.3390/s22239304

**Published:** 2022-11-29

**Authors:** Shengjun Guo, Kai Han, Wenbin Gong, Lu Li, Feng Tian, Xinglong Jiang

**Affiliations:** 1 Innovation Academy for Microsatellites of CAS, Shanghai 201204, China; 2 University of Chinese Academy of Sciences, Beijing 100049, China

**Keywords:** LEO satellite network, beam hopping, resource allocation, genetic algorithm

## Abstract

Low Earth Orbit (LEO) satellite communication networks have become an important means to provide internet access services for areas with limited infrastructure. Compared with the Geostationary Earth Orbit (GEO) satellites, the LEO satellites have limited on-board communication caching and calculating resources. Furthermore, the distribution of traffic requests is dynamically changing and uneven due to the relative movement between the LEO satellites and the ground. Therefore, how to schedule the multi-dimensional resources is an important issue for the LEO satellite communication networks. Beam-hopping is an efficient approach to improve the resource utilization by dynamically allocating time, power, and frequency according to the traffic requests. This paper proposes an efficient multi-dimensional resource allocation mechanism for beam-hopping in LEO satellite networks, which simultaneously satisfies the GEO interference avoidance. First, we construct the beam-hopping model of LEO satellites, and formulate the resource optimization problem. Second, we provide the weighted greedy strategy to determine the illumination pattern. In order to reduce the search space, the cells are clustered to non-interference clusters. Then, an improved genetic algorithm is provided to jointly allocate the communication resources. Finally, we construct various simulations to evaluate our proposed mechanism. Compared with the random-BH, polling-BH and traditional genetic algorithm, our algorithm achieves better performance in terms of both system throughput, access success rate, average delay and fairness between cells. The performance improvement is more significant in scenarios where traffic demand is unevenly distributed.

## 1. Introduction

With the soaring global demand for information increasing, satellite networks have become a powerful complement to terrestrial communications due to their wide coverage. As a technology expansion and integration of traditional aerospace and communication fields, the satellite Internet can easily achieve three-dimensional full coverage of land, sea and air including the poles and provide better support for the application of the Internet of Everything. There have been some high-throughput GEO projects [1,2,3] such as IPSTAR, Spaceway-3, “Zhongxing” and “Asia-Pacific” to meet the growing communication needs. However, GEO satellites have inherent shortcomings such as limited orbital resources, large delay, and high cost. Therefore, the deployment of LEO satellite constellations has gradually become an essential means to achieve the globalization, broadbandization and commercialization of communications [4].

In recent years, the world’s aerospace science and technology powers have proposed or implemented their own LEO satellite Internet constellation plans [5], which provides broadband Internet access service for areas with limited telecommunication infrastructure by launching hundreds or thousands of satellites. The satellites use phased array and high-throughput technology to provide ground users with ultra-low-latency broadband access with hundreds of megabytes of bandwidth. However, due to the small size and low power of LEO satellites, their onboard resources are severely limited. With the explosive development of large-scale LEO constellations, the competition for resources such as spectrum has become more intense. Meanwhile, due to the fast movement of LEO satellites, the sub-satellite coverage area and the electromagnetic environment change drastically, making both the available communication resources and terrestrial user requests extremely dynamic and complicated. Furthermore, according to the International Telecommunication Union (ITU), it is necessary for LEO satellites to control the interference to the GEO communication system within an acceptable level [6]. All the above constraints make it rather challenging to schedule the access resources of LEO satellites efficiently.

By flexibly controlling the antenna direction, frequency band, power and communication time slot, beam hopping (BH) can realize the on-demand allocation of communication resources for dynamic user requests, which is an effective way to solve the above challenges. So far, many scholars and enterprises have carried out a lot of research on beam hopping.

## 2. Related Work

The concept of beam hopping was first proposed in NASA’s project ACTS [7], which realizes beam hopping by switching the feed source through a switch. In 2007, the multi-beam satellite system Spaceway3 developed by Hughes Networks was equipped with a beam hopping module, which was the first instance of beam hopping being applied to an actual system and greatly improved system capacity and spectrum utilization through flexible radio resource assignment. Since then, many companies have scrambled to apply beam hopping technology to their high-throughput satellite systems, such as the Wideband Global Satellite (WGS) and Advanced Extremely High Frequency (AEHF) projects in the United States and the Wideband Internetworking Engineering Test and Demonstration Satellite (WINDS) system in Japan. Ref. [8] investigated the advantages of beam hopping and showed the performance and capacity improvement over non-hopped systems. Scholars have also proposed a new generation of high-throughput satellite communication system architecture based on beam hopping, and the hopping pattern design is jointly optimized by a convex optimization algorithm [9,10,11]. Li Guangxia’s team also sorted out the existing algorithms for satellite beam hopping resource allocation, and verified the good applicability of beam hopping in high-throughput systems [12]. However, the existing beam hopping research mainly focuses on GEO satellites. Due to the limited on-board resources and mobility of LEO satellites, the BH design scheme for GEO satellite systems cannot be directly applied to LEO satellite systems.

Some scholars have explored the adaptive improvement scheme of BH technology in the LEO satellite communication scenario. In [13], Liu introduced the concept of beam hopping into the LEO satellite system for the first time, and analyzed the effectiveness of the proposed iterative algorithm through system indicators such as throughput and delay. Ding proposed a satellite zoning coverage model and designed a clustering beam allocation method for the uneven traffic distribution in LEO satellite coverage area [14]. Tian proposed a greedy algorithm in the LEO system to allocate frequency and power to the beam, and evaluate the algorithm in terms of throughput and demand satisfaction rate [15]. References [16,17,18,19] constructed preliminary model of resource allocation with the goal of throughput or delay. However, the above studies only consider the resource allocation of single or some certain dimensions, which lacks the consideration of multi-dimensional resource allocation and does not propose a complete multi-objective optimization model. To address this issue, some scholars systematically study the joint allocation of multi-dimensional resources with the goal of maximizing throughput and minimizing transmission delay [20,21].

Recently, graph-based deep learning optimization, which can guarantee high performance with low complexity, has received great attention and has been applied in communication networks [22]. Deep learning methods can capture the spatial information hidden in the network topology and work well for high dynamic scenes, thus providing new ideas for satellite traffic prediction [23] and resource allocation [24,25,26]. For example, Ref. [27] applied deep reinforcement learning in a beam-hopping problem to decide the illumination pattern and bandwidth allocation and achieve superior performance; however, the problem scale is small. Ref. [28] has a relatively large scale, but only focuses on cell selection.

In addition to certain limitations in problem scale and resource dimension, the above studies also ignore some inherent problems. The existing work often only consider the constraints of total on-board bandwidth and power, ignoring the coupling relationship between them when considering interference in practical scenarios. On the one hand, many studies only limit co-channel interference (CCI) through physical isolation, which prevents adjacent hotspots from being served simultaneously. On the other hand, few studies have considered the problem of interference avoidance for GEO systems. In fact, the frequency band allocated to the LEO beam determines the limit of its transmitting power. In view of the above problems, in the scenario of sharing spectrum with GEO, we model the problem of beam hopping resource allocation as a multi-objective optimization problem, and propose a mechanism to efficiently and flexibly allocate the time slot, power and bandwidth. The main contributions of this paper are as follows:

1. In the multi-beam LEO satellite communication system, we model the joint allocation of communication time slot, frequency and power three-dimensional resources as a multi-objective optimization problem. While considering both the inter-beam CCI and interference avoidance to the GEO system, we achieve dynamic beam hopping according to different distribution of service request traffic in space and time.
2. We propose a flexible and efficient resource allocation mechanism in this paper. Firstly, we comprehensively measure the amount and urgency of traffic demand of all cells, and determine the illumination pattern through the weighted greedy strategy. Then, the interference clustering operation is performed based on the antenna model gain curve, and the lighting cells of the current time slot are clustered. Finally, the joint allocation of the bandwidth and power of the cells in each cluster is completed through the improved genetic algorithm or according to the constraint map.
3. In the scenario of even and uneven traffic distribution, a series of simulations are carried out under different traffic intensities to evaluate the performance of our proposed algorithm. Compared with random BH, polling BH and traditional genetic algorithm, the simulation results show that our proposed method can achieve higher throughput and access success rate, and reduce the average delay of all data packets while ensuring service fairness between cells.

The remainder of this paper is organized as follows: Section 3 describes the system scenario and expounds the difficulty of solving the problem, and then the problem model and formulation are given. Section 4 elaborates the allocation mechanism and the specific implementation of our proposed algorithm. Section 5 shows some simulations and analysis to demonstrate the effectiveness of our proposed algorithm. Section 6 summarizes this article.

## 3. System Model and Problem Formulation

### 3.1. Scenario Description

In this paper, we consider a mega-constellation of Ka-band LEO satellites deployed at 1050 km from earth. There is a GEO high throughput satellite above one of the orbits of the LEO system and within the coverage area of LEO satellite, there are some GEO ground stations which share the same spectrum with LEO systems. Each LEO satellite is equipped with a steerable phased array antenna, a fixed signaling antenna and a regenerative telecommunication transceiver. The fixed signaling beam can cover the entire satellite footprint and is responsible for the signaling transmission including the uploading of ground user request. The steerable multi-beam phased array antenna can generate *K* spot beams for transmitting traffic data, which can be directed to any area of ±50°, and the satellite can flexibly and dynamically allocate on-board power and bandwidth to these spot beams.

**Figure 1:**
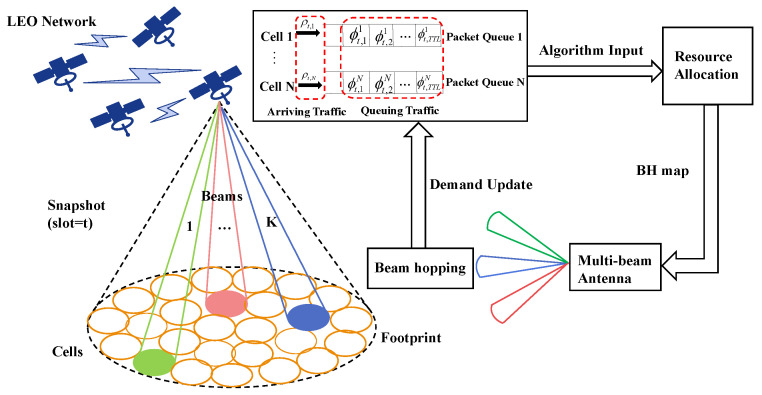
Schematic diagram of the beam hopping scenario.

There are N(N>K) cells in each satellite footprint, each spot beam can cover one cell and all sub-satellite coverage areas can be served by *K* beams in a time-division multiplex manner. In this paper, we divide time into time-segments and assume that the relative position of the LEO satellite and the ground are fixed within the time-segment. Each time-segment is further divided into T time slots, which is the minimum time unit for beam dwelling. The satellite receives the terminal request through the signaling beam, then the service spot beam can hop from one cell to another according to the traffic demand distribution and allocate the beam resources. Assuming that the satellite can provide *N* queues to store the arriving traffic of each cell and each request can only survive for $T_{ttl}$ time due to the limited queue length. $T_{ttl}$ is defined as Time To Live, which is an integer multiple of the time slot length, and packets that cannot be satisfied within this time will be discarded. The whole scenario is shown in Figure 1, and in this paper, we mainly focus on the downlink resource allocation. In the following context, to avoid confusion, a lit cell and a serving beam are essentially the same concept.

In the beam hopping scenario of this paper, the resources that need to be flexibly allocated are communication time slots, bandwidth and power. According to the real-time traffic demand distribution on the ground, we first determine which *K* cells are illuminated by the *K* beams in each time slot to serve them, which is called an illumination pattern in beam hopping. Each beam can flexibly allocate the bandwidth and power. The total bandwidth on the satellite is $B_{tot}$, which is divided into *m* sub-bands of equal bandwidth, and each beam can use one or more consecutive sub-bands. The total power available on the satellite is denoted as $P_{tot}$, which means the sum of the power used by all spot beams lit in the same time slot cannot exceed this limitation. For the protection of higher-priority GEO systems, the transmitting power of LEO satellite beams will be limited to ensure that the signal power received by the GEO ground stations is controlled within the interference threshold.

There are two main challenges for resource scheduling problem in BH scenario. On the one hand, the distribution of satellite terminals in different ground areas is different and the bandwidth requirements are also different, thus facing an extremely uneven space-time distribution of service access requests. Due to the rapid movement of satellites, the terminal needs to switch frequently between different beams and the satellite nodes. On the other hand, when allocating three-dimensional resources of time slot, frequency and power at the same time, the search space is very large, and the allocation of resources in each dimension has strong coupling, so it cannot be decoupled and solved separately. More specifically, assuming that there are *N* cells under the coverage area of a single satellite, and at the same time slot we can select *K* cells to be simultaneously illuminated, so there are $C_{N}^{K}$ illumination patterns for each time slot. Moreover, each beam can use a continuous segment of *m* sub-frequency-bands, which means there are $\frac{m(m+1)}{2}$ types of band allocation schemes for each beam to select. Therefore, consider a time-segment containing *T* time slots, the searching space of the entire beam hopping allocation strategy is ${\left(C_{N}^{K}\times {\left(\frac{m(m+1)}{2}\right)}^{K}\right)}^{T}$, which makes it difficult to find the global optimal solution.

**Figure 2:**
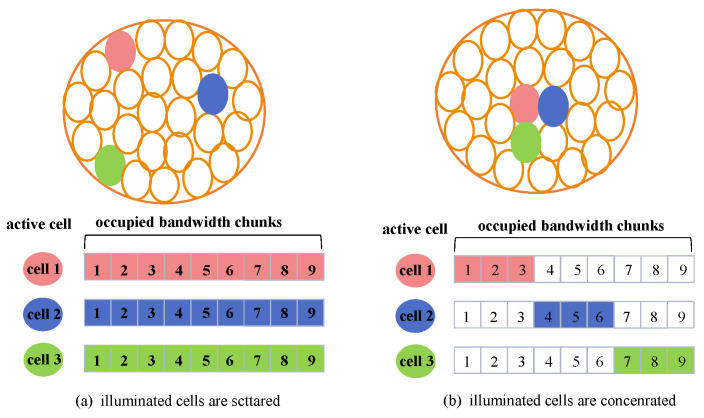
Schematic diagram of strong coupling between illumination pattern and bandwidth allocation. (**a**) shows a situation where the lighting cells are scattered, and each cell can utilize the full bandwidth. (**b**) shows a situation where the lighting cells are concentrated, and each cell needs to isolate the bandwidth to eliminate interference.

In order to illustrate the strong coupling relationship between three dimensional resource allocation more intuitively, we give an example as shown in Figure 2. Assuming that we can light three cells at the same time slot and each serving spot beam can use 9 consecutive equal-width sub-bands. Consider two different actual scenarios, in Figure 2a, the distribution of hotspot areas with large traffic demand is relatively scattered, the cells that are lit at the same time are geographically dispersed, and there is almost no co-frequency interference, so each beam can use the full frequency band. While in Figure 2b, hot spots are geographically concentrated and all three adjacent cells have great access requirements. Once they are lit at the same time with a full band, co-channel interference will become very serious and effective communication will not be possible. In this case, three consecutive sub-bands are allocated to adjacent beams, respectively, which can not only meet the needs of simultaneous communication of hot spots, but also avoid mutual interference. Moreover, depending on the selected frequency band, the transmitting power limit of the beam will be different due to the interference avoidance requirements of the GEO ground station.

### 3.2. Link Calculation Model

The useful power received by the user terminal located under the beam can be calculated by the following formula:

(1) $$\left[P_{R}\right]=\left[P_{T}\right]+\left[G_{T}\right]+\left[G_{R}\right]-\left[L\right]$$

in which $P_{T}$ is the transmitting power of the satellite beam, $G_{T}$ is the transmitting gain of the satellite antenna, $G_{R}$ is the antenna receiving gain of the user terminal, *L* is the transmission loss, which can be calculated as follows:

(2) $$L=L_{fsl}+L_{rsl}+L_{aml}+L_{aa}+L_{pl}$$

where $L_{fsl}$ is the free space loss, $L_{rsl}$ is the feeder loss of the receiver, $L_{aml}$ is the antenna misalignment loss, which includes the antenna pointing loss and polarization loss, $L_{aa}$ is the fixed atmospheric loss, and $L_{pl}$ is the ionospheric loss. The main component of *L* is the free space loss, and the specific calculation equation is

(3) $$\left[L_{fsl}\right]=20\mathrm{lg}\left(f\right)+20\mathrm{lg}\left(r\right)+32.4$$

in which the unit of frequency *f* is MHz, and the unit of distance between transmitter and receiver *r* is km. The calculation of polarization loss can refer to the model in document ITU R P. 618-13 [29], and the calculation of atmospheric loss and ionospheric loss can refer to document ITU R P.676-12 [30] and ITU R P.531-14 [31], respectively. The feeder loss of the receiver and antenna pointing loss are set as constant parameters, taking 0.5 dB and 2 dB, respectively, according to engineering experience.

The noise power received by the user terminal can be calculated by Equation (4):

(4) $$P_{N}=kT_{S}B_{N}$$

where k=−228.6012 dBW/ (Hz·K${}^{-1}$) is the Boltzmann constant, $T_{S}$ is the equivalent system noise temperature, and $B_{N}$ is the equivalent noise bandwidth. After determining the useful signal power and noise power at the receiver input, we can then obtain the input signal-to-noise ratio

(5) $$\frac{C}{N}=\frac{P_{R}}{P_{N}+I_{R}}$$

It is worth noting that we regard the interference as a part of the noise. In Equation (5), $I_{R}$ is the interference power received by the user terminal under the coverage of the current beam, which is mainly the co-channel interference from other beams that lit at the same time slot, and the specific calculation method will be given in the interference model.

### 3.3. Antenna Model

The antenna radiation pattern is crucial in interference analysis and is a key input for system simulation experiments. To facilitate frequency coordination and interference assessment in different frequency bands, the ITU has issued some radiation pattern reference recommendations for earth station and satellite antennas such as ITU-R S.465-6 [32], ITU-R S.672-4 [33], ITU-R S.1428-1 [34], and ITU-R S.1528 [35]. In this paper, in order to simplify the problem, we adopt a unified antenna model on both the satellite and the ground terminals without loss of generality, in which the beam gain can be calculated by the following equation [36,37]:

(6) $$G\left(\theta \right)=G_{0}{\left[\frac{J_{1}\left(u\left(\theta \right)\right)}{2u(\theta )}+36\frac{J_{3}\left(u\left(\theta \right)\right)}{u{\left(\theta \right)}^{3}}\right]}^{2}$$

where θ represents the off-axis angle, which means angular position of the terminal user from the beam center with respect to the satellite. $G_{0}$ is the maximum antenna gain defined as $G_{0}=\eta N^{2}\pi^{2}/{\theta_{3dB}}^{2}$, in which η is the antenna efficiency, *N* is a constant related to the field distribution of the antenna radiation pattern, $\theta_{3dB}$ is the antenna 3dB gain angle. $J_{1}\left(\cdot \right)$ and $J_{1}3\left(\cdot \right)$ are the first-order and third-order Bessel functions of the first kind, respectively. $u\left(\theta \right)=2.07123\sin\theta /\sin\theta_{3dB}$. The 3dB gain angle of the satellite transmitting antenna can be calculated as follows:

(7) $$\theta_{3dB}=\frac{180^{\circ }}{\pi }\arctan\left(\frac{R}{H}\right)$$

in which *H* represents the satellite orbit height, *R* denotes the single beam radius. Once the 3 dB angles are obtained, we can determine the antenna radiation patterns of the satellite and terrestrial terminal through Equation (6). In this paper, the parameters related to the antenna radiation pattern are set as shown in Table 1:

Substituting the above parameters into the model for the simulation, we can obtain the antenna radiation pattern of the satellite and the terrestrial terminal.

### 3.4. Interference Model

There are two main types of interference considered in our scenario: (1) co-channel interference between LEO beams; and (2) interference from LEO beams to GEO ground stations. In the following subsections, we will give the model and analysis, respectively, for the two types of interference.

#### 3.4.1. Co-Channel Interference between LEO Beams

**Figure 3:**
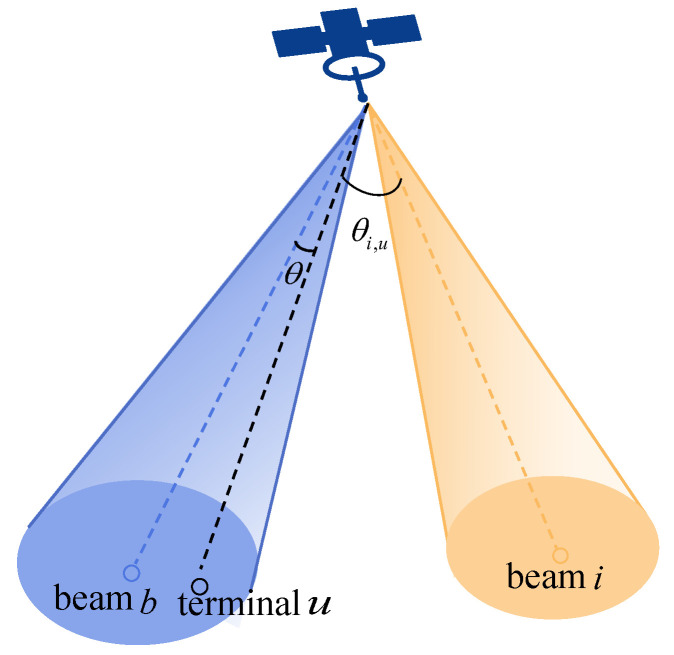
Schematic diagram of co-channel interference between beams.

The schematic diagram of co-channel interference between beams is shown as Figure 3.

In Figure 3, beam *b* and beam *i* are lit in the same time slot and their allocated frequency bands overlap with each other, then the interference from beam *i* received by terminal *u* in beam *b* is calculated as follows.

(8) $$I_{i,u}^{CO}=P_{i}G_{i}\left(\theta_{i,u}\right)G_{u}\left(\theta \right)/L$$

where $P_{i}$ is the transmitting power of beam *i*, $G_{i}\left(\theta_{i,u}\right)$ is the gain of beam *i* in the angular direction $\theta_{i,u}$ deviating from the main axis, $G_{u}\left(\theta \right)$ is the receiving gain of terminal *u*, and *L* is the transmission loss.

In this paper, in order to simplify the model without losing generality, we assume that the terminals covered by the beam are all located at the center of the beam, that is, in the above formula, $G_{u}\left(\theta \right)$ is a constant equal to peak gain of the receiving antenna, and in actual calculation, $\theta_{i,u}$ should be substituted into $\theta_{i,u}+\theta$ as shown in the figure.

**Figure 4:**
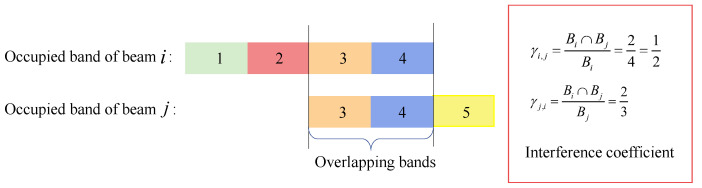
Illustration diagram of interference coefficient calculation.

The total interference to the terminal *u* is the sum of the interference from all the beams illuminated at the same time slot, which is denoted as

(9) $$I_{tot,u}=\sum_{i}\gamma_{u,i}I_{u,i}^{CO}$$

where *i* represents all surrounding cells illuminated by co-frequency and co-polarized beams, and $\gamma_{u,i}$ is the interference coefficient of the two cells, which is defined as the ratio of the overlapped sub-bands of the two cells to the bandwidth of the total used frequency band as sketched in Figure 4.

As shown in Figure 4, beam *i* and beam *j* occupied four and three sub-bands, respectively, and the overlapping part contains two chunks. For beam *i*, the interference coefficient is 1/2, and for beam *j*, the interference coefficient is 2/3.

#### 3.4.2. Interference from LEO Beams to GEO Ground Stations

Countries around the world have gradually recognized the strategic significance and important values of satellite frequency and orbit resources in the development of space economy, and declared a large amount of satellite network applications to ITU to seize the frequency and orbit resources. At present, emerging NGSO communication constellation systems represented by O3b, Starlink, OneWeb, Telesat, Kuiper, etc., plan to use very concentrated frequencies, mainly Ku/Ka/Q/V. Traditional Geostationary Satellite Orbit (GSO) satellite communication systems also mainly use Ku/Ka frequency band resources, and a large number of systems are already operating in orbit [38]. According to Article 22 of the ITU “Radio Regulations” [39], when using the Ku/Ka frequency bands, the communication systems of the NGSO fixed-satellite service and the satellite broadcasting service shall not cause unacceptable interference to the GSO satellite communication system, and shall not seek the protection of the GSO satellite communication system.

The interference power from LEO spotbeam serving cell *i* to a GEO earth station *e* is denoted by

(10) $$I_{i,e}=\delta_{i,e}P_{i}G_{t}^{l}\left(\theta_{i,e}\right)G_{r}^{g}\left(\theta_{e}\right)/L_{e}^{l}$$

where $\delta_{i,e}\in \left\{0,1\right\}$ is a binary variable that indicates whether the frequency band occupied by the LEO spot beam and the GEO ground station overlaps, and $\delta_{i,e}=1$ means there is overlap, otherwise $\delta_{i,e}=0$. $P_{i}$ represents the transmitting power of the LEO spotbeam *i*, $G_{t}^{l}\left(\theta_{i,e}\right)$ is the transmitting antenna gain of the LEO satellite, and $G_{r}^{g}\left(\theta_{e}\right)$ denotes the receiving antenna gain of the GEO earth station.

To simplify the model, we only consider the free space loss in the transmission loss, which is $L_{e}^{l}=4\pi d_{e}^{l}/\lambda$, where $d_{e}^{l}$ is the distance between LEO satellite and the GEO earth station and λ is the carrier wavelength.

The interference threshold of the LEO satellite beam to the GEO ground station is set as $I_{th}$. On the one hand, when $I_{i,e}>I_{th}$, it is considered that the LEO beam produces unacceptable interference to the ground station, and the beam should be turned off at this time, and other LEO satellites should be selected to serve the cell in the case of multiple coverage. On the other hand, the protection of GEO ground stations limits the maximum power transmitted in each subband.

**Figure 5:**
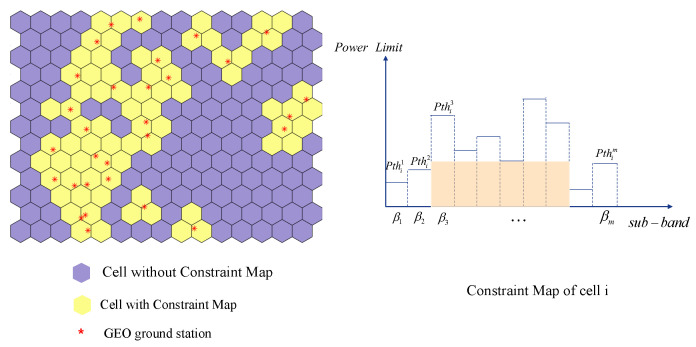
Illustration diagram of the Constraint Map.

More specifically, for each sub-band $\beta_{m}$, it corresponds to a set of ground stations that share the same frequency band, which is denoted as $E_{m}=\left\{e_{1},e_{2},\cdots e_{\xi }\right\}$. For a certain cell (beam), the off-axis angles of the ground stations in the set *E* regarding to the beam are different, but they all meet the same threshold $I_{th}$. According to Equation (10), the maximum transmitting power of the beam with regard to each ground station can be obtained, and the set of them is denoted as $P_{i}^{\beta m}=\left\{P_{1},P_{2},\cdots P_{\xi }\right\}$, in which the minimum power is the corresponding threshold transmitting power when the beam *i* uses the subband $\beta_{m}$, that is $Pth_{i}^{\beta m}=\min P_{i}^{\beta m}$. In this paper, we define this constraint relationship as the Constraint Map of the cell, which is shown in Figure 5.

### 3.5. Problem Formulation

After allocating bandwidth and corresponding power to the cells, the channel capacity of cell *i* in the time slot *t* can be calculated according to Shannon’s formula as follows:

(11) $$C_{t}^{i}=\chi_{t}^{i}B_{t}^{i}\log\left(1+{\frac{C}{N}}_{t}^{i}\right)$$

in which $\chi_{t}^{i}$ is a binary variable used to indicate whether cell *i* is illuminated in the time slot *t*, and $\chi_{t}^{i}=1$ indicates illumination, otherwise $\chi_{t}^{i}=0$. $B_{t}^{i}$ is the allocated bandwidth, and ${\frac{C}{N}}_{t}^{i}$ is the signal-to-noise ratio obtained by Equations (4), (5), (8) and (9). The traffic demand of cell *i* at time slot *t* is $D_{t}^{i}$. The throughput of a cell is defined as the minimum of demand and capacity, i.e., $Thro_{t}^{i}=\min\left\{C_{t}^{i},D_{t}^{i}\right\}$.

While improving the system throughput, we hope that the delay of each service request is as small as possible to ensure the quality of service. As mentioned in Section 3.1, there are *N* queues on the satellite to store the request data for each cell, and the traffic stored in the queue is denoted by $D_{t}=\left\{D_{t}^{i}\left|i\in N\right.\right\}$, in which $D_{t}^{i}$ represents the stored traffic of cell *i* in time slot *t*. Since the waiting time of each data packet in the queue is different, the demand can be further subdivided into $D_{t}^{i}=\sum_{l=1}^{T_{ttl}}\phi_{t,l}^{i}$, where $\phi_{t,l}^{i}$ denotes the number of packets that have been waiting in queue *i* for *l* time slots. Packets that are not responded to within $T_{ttl}$ time will be discarded and regarded as access failures. The average waiting delay of data packets in the queue of cell *i* in time slot *t* is

(12) $$\tau_{t}^{i}=\frac{\sum_{l=1}^{T_{ttl}}l\cdot \phi_{t,l}^{i}}{\sum_{l=1}^{T_{ttl}}\phi_{t,l}^{i}}$$

In the entire beam hopping period, the number of data packets requested in each cell is denoted as $n_{total}^{i}$, the number of packets that dropped due to timeout is $n_{fail}^{i}$, and the access success rate can be calculated by the following equation:

(13) $$SucRate=\frac{\sum_{i=1}^{N}\left(n_{total}^{i}-n_{fail}^{i}\right)}{\sum_{i=1}^{N}n_{total}^{i}}$$

The average response delay of all data packets in the entire beam hopping period is

(14) $$\tau_{average}=\frac{\sum_{i=1}^{N}\sum_{l=1}^{T_{ttl}}l\cdot \phi_{l}^{i}}{\sum_{i=1}^{N}\sum_{l}^{T_{ttl}}\phi_{l}^{i}}$$

in which $\phi_{l}^{i}$ denotes the number of packets in cell *i* that waited for *l* slots before being responded during the entire beam hopping period.

While minimizing the delay, we want to ensure the fairness of service in each cell, which can be characterized by minimizing the variance of the average waiting delay of data packets between cells as follows:

(15) $$Fair=\mathrm{var}\left(\tau_{i}\right)=\mathrm{var}\left(\frac{\sum_{l=1}^{T_{ttl}}l\cdot \phi_{l}^{i}}{\sum_{l}^{T_{ttl}}\phi_{l}^{i}}\right)$$

In summary, we can model the entire beam hopping assignment problem as an optimization model as follows:

(16) $$\begin{array}{c} P1:\max\sum_{t=1}^{T}\sum_{i=1}^{N}Thro_{t}^{i}\\ P2:\max SucRate\\ P3:\min\tau_{average}\\ P4:\min Fair\\ s.t.\\ C1:B_{t}^{i}\subseteq \left\{\beta_{1},\beta_{2},\cdots ,\beta_{m}\right\}\\ C2:P_{t}^{i}\leq Pth_{i}^{\beta m}\;\;\;\;\;\;\;\;\;\;\;\;\;\;\;\;\;\;\;\forall \beta_{m}\in B_{t}^{i}\\ C3:\sum_{i=1}^{N}P_{t}^{i}<P_{tot}\;\;\;\;\;\;\;\;\;\;\;\;\;\forall i\in N\\ C4:\sum_{i=1}^{N}\chi_{t}^{i}\leq K\;\;,\;\;\;\;\chi_{t}^{i}\in \left\{0,1\right\} \end{array}$$

In the above optimal problem, $\chi_{t}^{i}$, $B_{t}^{i}$ and $P_{t}^{i}$ are the variables to be optimized, which are defined as the illumination pattern and bandwidth and power allocation of each beam at each time slot. P1 and P2 aim to achieve maximum system throughput and access success rate during the entire beam hopping period. P3 and P4 ensure the fairness of service between cells while minimizing the average delay of all data packets. C1 requires each beam to occupy one or more contiguous subbands. C2 indicates the constraint for the maximum transmitting power per subband of each beam for the protection of GEO terrestrial stations. C3 ensures that the sum of the allocated power of all illuminated beams cannot exceed the total available power onboard. C4 indicates that at most *K* beams can be lit in the same time slot.

It is not difficult to find that the above formula is a non-convex and non-linear problem and it contains a binary variable $\chi_{t}^{i}$. Since we divide the total available band into *m* equal-bandwidth subbands, the bandwidth allocation part involves integer programming. Therefore, this optimization problem is also NP-hard and we cannot obtain the optimal solution in polynomial time.

## 4. Resource Allocation Mechanism

In the case of large decision spaces, heuristic or intelligent optimization methods that obtain high-quality solutions at acceptable computational cost are more attractive in practical engineering environments. To deal with this problem, we decompose the problem into two sub-problems, which are the determination of illumination pattern and the joint allocation of bandwidth and power. In the first stage, we comprehensively consider the demand and urgency (whether it faces the dilemma of being discarded due to timeout) of each cell, and determine the illumination pattern of each time slot in real time based on a weighted greedy strategy. In the second stage, we adopt interference clustering and improved the genetic algorithm for the joint allocation of bandwidth and power to the active beams.

### 4.1. Weighted Greedy Strategy for Illumination Pattern

As described in Section 3.5, the traffic demand of each cell can be further expanded to $\left\{\phi_{t,1}^{i},\phi_{t,2}^{i},\cdots ,\phi_{t,l}^{i},\cdots ,\phi_{t,T_{ttl}}^{i}\right\}$ according to different waiting time. Since we set the expiration time limit of data packets as $T_{ttl}$ in this paper, we define the demands that have waited for more than 4/5 $T_{ttl}$ as urgent, which are denoted as $d_{u,t}^{i}$ and are prone to fail to access due to timeout if not satisfied in time. The rest of the requirements are non-urgent as they can wait for an additional period of time, which is denoted as $d_{nu,t}^{i}$. We first normalize the two types of demands of each cell by the following formula:

(17) $$nor\_d_{*,t}^{i}=\frac{d_{*,t}^{i}}{\sum_{i=1}^{N}d_{*,t}^{i}}$$

in which ∗=u represents urgent demands and ∗=nu represents non-urgent demands. In order to seek a trade-off between the total amount and urgency of the demand in each cell, we take the weighted sum of the two normalized demands to obtain the current traffic request of each cell as follows:

(18) $${\tilde{d}}_{t}^{i}=\omega_{1}*nor\_d_{u,t}^{i}+\omega_{2}*nor\_d_{nu,t}^{i}$$

where $\omega_{1}$ is the weight of urgency, and the higher the weight, the more the system tends to respond to requests that are about to expire, ensuring a high access success rate. $\omega_{2}$ is the weight of demand amount, the higher the weight, the more the system tends to allocate beam resources to the cell with the most traffic demand.

After calculating the weighted demand of each cell in each time slot by the above formula, we use the greedy strategy to select the *K* cells with the largest $d_{t}^{i}$ to form the illumination pattern of the current time slot. The Weighted Greedy Strategy (WGS) that selects the illuminated cells in a time slot is summarized in Algorithm 1. After completing the joint allocation of bandwidth and power for the current time slot in the second stage, update the traffic demand of each cell and determine the illumination pattern of the next time slot according to the WGS again, then repeat the process and iterate until the end of the entire beam hopping cycle.
**Algorithm 1** WGS for illumination pattern determination.**Input:** collection of cells {N}, set of demand of all cells $D_{t}=\left\{D_{t}^{i}\left|i\in N\right.\right\}$**Output:** the set of illuminated cells Φ1:Initialize Φ=Ø, Count=0;2:**for** i=1 to N **do**3:    count the $d_{u,t}^{i}$ and $d_{nu,t}^{i}$;4:**end for**5:Normalize two types of demand: $nor\_d_{*,t}^{i}=d_{*,t}^{i}/\sum_{i=1}^{N}d_{*,t}^{i}$;6:Obtaion weighted demand: ${\tilde{d}}_{t}^{i}=\omega_{1}*nor\_d_{u,t}^{i}+\omega_{2}*nor\_d_{nu,t}^{i}$;7:**while**Count<K**do**8:    $i=\arg\max{\tilde{d}}_{t}^{i},i\in \left\{N\right\}$9:    Add *i* to Φ and reomve *i* from {N}10:    Count=Count+111:**end while**

### 4.2. Interference Clustering + Improved Genetic Algorithm

For the joint allocation of bandwidth and power, many scholars have adopted genetic algorithms to solve them. However, as mentioned in Section 3.1, with the increase of the number of beams, the search space for all beams to be optimized together is very large, which will lead to slow convergence of the algorithm and cannot obtain a satisfactory solution.

In addition, in order to avoid CCI, many articles set a distance threshold. More specifically, it is generally considered that there is no interference if the distance between two cells exceeds four times the radius of the cell, i.e., non-adjacent cells. On the one hand, it is inaccurate to measure interference based on distance. From the antenna model in Section 3.3, we can easily discover that the change of gain with an off-axis angle is not monotonic, and the distance between two cells is not in a one-to-one correspondence with their off-axis angle. On the other hand, it is unrealistic to restrict adjacent cells from being illuminated at the same time slot in order to avoid CCI, which will cause the beam to hop back and forth between two adjacent hot spots.

#### 4.2.1. Interference Clustering

**Figure 6:**
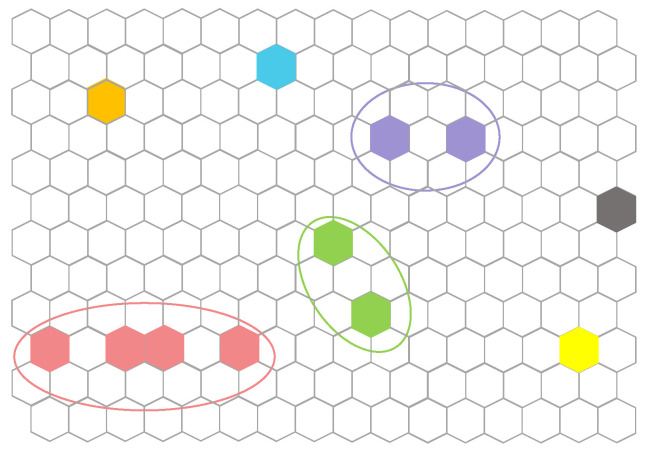
An example of interference clustering result.

To address these issues, we propose interference clustering based on the antenna model. More specifically, after determining the *K* active beams in current time slot, the off-axis angle between any two illuminated cells relative to the satellite can be calculated based on the cell center and the satellite position, and the corresponding antenna gain can be further obtained according to the gain curve. Set the interference threshold according to the gain and divide the *K* illuminated cells into independent clusters, and we consider that there is no CCI between clusters. Figure 6 shows the clustering of illuminated cells in a certain time slot. Within each cluster, if there are multiple cells, we adopt the improved genetic algorithm (IGA) to complete the joint allocation of bandwidth and power, and the search space becomes smaller due to the reduction of the number of beams. If the cluster only contains one single cell, we find the scheme with the largest capacity according to its Constraint Map as mentioned in Section 3.4.2.

#### 4.2.2. Joint Allocation of Bandwidth and Power

(A) Multi-cell Assignment Based on IGA

When there are multiple cells in the cluster, considering the CCI between cells, we propose an improved genetic algorithm to search for the frequency and power allocation scheme with the goal of maximizing the total throughput of each cell. More specifically, we regard each resource allocation scheme as a chromosome, and the genes contained in the chromosome include all beam-related resource elements (i.e., bandwidth and power in this paper) of the system. By performing selection, crossover, mutation and elite retention operations on chromosomes, a high-quality resource allocation scheme is obtained.

It is worth noting that, in order to reduce the length of chromosomes, we only encode the frequency band assigned to each beam, and the corresponding power constraints and adjustments are reflected in the calculation of the fitness function. In this paper, the total available band is divided into 10 equal bandwidth sub-bands numbered 1–10. The frequency band scheme allocated by each active beam is represented by the number of the starting and ending sub-band, and each number needs to be represented by a 4-bit binary number. For example, if beam *i* occupies the frequency band numbered 4–9, its corresponding code is 01001001. The allocation scheme for each beam requires an 8-bit code representation, thus the chromosome length corresponding to each resource allocation scheme is 8∗n if there are *n* cells in the cluster.

When calculating the fitness function, we firstly obtain the maximum transmitting power of each beam according to the frequency band of each beam and the power constraint relation of the frequency calculated in Section 3.4.2. We consider the CCI among all cells in the cluster, calculate the capacity of each beam, and then obtain the throughput of each beam according to the demand of each cell. Take the sum of the throughputs of all cells in the cluster as the fitness of the scheme. According to the value of fitness, half of the individuals in the population are selected by the roulette method to be retained to the next generation. When performing crossover and mutation operations, the power is updated according to the constraints, and the other half of the offspring individuals are obtained.

The convergence criteria of the algorithm is as follows: denote the best solution in the set of resource allocation solutions generated by the $g_{th}$ iteration as best, and the sum of all beam throughputs obtained by this solution is $thro_{best}$, and compare it with the average value of the previous *l* results, if it is less than a certain threshold, the resource allocation algorithm is considered to have converged, and the iterative loop is exited. Iteration should also stop when the set maximum number of iterations is reached. The parameter settings related to the genetic algorithm are shown in Table 2.

In order to guide the algorithm to search for a high-quality feasible solution region, speed up the algorithm convergence, and reduce the number of iterations, we embed an optimization control strategy in the algorithm design. On the one hand, we reduce the randomness of the crossover and mutation operators. If the beam capacity decreases after crossover and mutation, we continue to perform operations, and the solution with an optimized capacity is reserved. On the other hand, we adopt an elite retention strategy, that is, pick out the best-performing individual in the previous generation population and replicate it in the next generation population. Compared with the traditional genetic algorithm, the improved algorithm with the added control strategy is less likely to fall into the local optimal solution, and the convergence speed is significantly faster.

**Table 2:** Parameter settings related to the genetic algorithm.

Parameter	Value
Population scale	200
Selelct probability	0.5
Crossover probability	0.7
Mutation probability	0.01
Iteration times	50

(B) Single-cell Assignment Based on Constrain Map

If there is only one single cell in the cluster, the CCI between cells does not need to be considered, and the only constraint is the power constraint on the GEO ground station. As illustrated in Section 3.4.2, for a certain cell, each sub-band corresponds to a power limit when used alone, which forms a Constraint Map. We find out the combination of frequency bands that maximizes the capacity of the cell directly according to the constraints. In the example shown in Figure 5, the total available frequency band is divided into 10 equal bandwidth sub-bands denoted as $\beta_{1}$ to $\beta_{10}$ (It is worth noting that the bandwidth of each sub-band can be different, and we use equal bandwidth in this paper to simplify the model). The maximum transmitting power over each sub-band for cell *i* is denoted as $p_{th}^{1}$ to $p_{th}^{10}$. Since cell *i* achieves maximum communication capacity when the beam occupies $B_{i}=\left\{\beta_{3},\beta_{4},\cdots ,\beta_{8}\right\}$ and the corresponding power is $P_{i}=p_{th}^{6}$, therefore, the optimal resource allocation scheme for cell *i* should be $B_{i}=\left\{\beta_{3},\beta_{4},\cdots ,\beta_{8}\right\},P_{i}=p_{th}^{6}$. The joint allocation mechanism of bandwidth and power is summarized in Algorithm 2.
**Algorithm 2** Joint allocation mechanism of bandwidth and power.**Input:** Illuminated cells in current time slot: {N}, demand of illuminated cells: $D_{t}=\left\{D_{t}^{i}\left|i\in N\right.\right\}$, onboard available resources: $B_{tot}$, $P_{tot}$, sub-bands: $\left\{\beta_{1},\beta_{2},\ldots ,\beta_{m}\right\}$, constraint between power and band: $\{Pth_{i}^{\beta m}\}\;\;$**Output:** $B_{i}$ and $P_{i}$ for each illuminated cell1:Perform interference clustering according to Section 4.2.1 and the number of cells in each cluster is *n*;2:**if**n>1**then**;3:    Population initialize4:    **for** t=1 to iteration time **do**5:        Calculate fitness according to Section 4.2.2 (A)6:        Record elite with best fitness7:        Perform selection, coding, crossover, mutation and decoding with optimal control considering $\{Pth_{i}^{\beta m}\}\;\;$;8:        Replace the least fit individuals with elite;9:    **end for**10:    Calculate $C_{i}$ by Equation (11);11:**else**12:   Select $B_{i}=\left\{\beta_{a},\ldots ,\beta_{b}\right\}$ and $P_{i}=min\left\{Pth_{i}^{\beta m},m\in \left(a,b\right)\right\}\;\;$ according to Section 4.2.2 (B) based on Constraint Map shown as Figure 5;13:**end if**14:Recalculate $C_{i}$ considering the CCI of all cells according to Section 3.4.1;15:**return** allocation scheme $B_{i}$, $P_{i}$ and capacity $C_{i}$;

After completing the resource allocation to cells in all clusters, we comprehensively consider the CCI among all the illuminated cells, and calculate the transmission capacity of all cells again. According to the traffic demand of each cell and the provided communication capacity, we update the traffic demand in the next time slot and continue to determine active beams and perform resource allocation until the end of the beam hopping period.

## 5. Simulation and Results

In this section, we carried out various simulations to verify the effectiveness of our proposed algorithm in LEO beam hopping scenario. In this paper, there is a mega-constellation of Ka-band LEO satellites deployed at 1050 km and the cell illuminated by each beam is a regular hexagon with a side length of 40 km. In order to form a complete beam hopping map, the following two reasonable assumptions should be accepted due to the duration of a transmission cycle is short enough: (1) The position of the LEO satellite relative to the terrestrial cell is static during a beam hopping period. (2) The channel state is constant within one beam hopping transmission period. Above the orbit of the LEO system, there is a GEO high throughput satellite sharing the same spectrum, and some GEO ground stations are scattered in the footprint of LEO satellites.

### 5.1. Simulation Settings

We assume that a time segment, i.e., a beam hopping cycle, has 256 time slots and the length of each time slot is 10ms. Service requests generated by terrestrial cells obey Poisson distribution. In order to avoid congestion caused by excessive traffic, the traffic waiting for more than 50 time slots will be discarded, that is $T_{ttl}=50T_{slot}$. The main simulation parameters are listed in Table 3.

The following performance metrics are presented to evaluate the performance of the LEO beam hopping communication system.

- Throughput:
  The system throughput is defined as the average throughput of 12 beams over the entire BH cycle.
- Access Success Rate:
  The access success rate is defined as the ratio of the number of packets served of all cells in the BH cycle to the demand generated.
- Average Delay:
  The average delay is defined as the average queuing time for all served packets.
- Fairness:
  The fairness between cells is defined as the variance of the average delay of the packets served by each cell.

In this paper, we compare our proposed algorithm with three different BH schemes, and a brief description of the comparison algorithms is as follows.

(1) The polling beam hopping (Polling-BH): divide all cells into 12 uniform blocks, and each beam will serve the cells in a block in turn in a fixed order. Due to the inherent geographic isolation, each beam can use the full bandwidth.

(2) Random beam hopping (Random-BH): the random hopping method randomly selects K cells from N cells for service at each time slot. Moreover, the bandwidth of each beam is also chosen randomly from m(m+1)/2=55 available bandwidth allocation schemes at each time slot.

(3) Genetic algorithm without interference cluster (GAWIC-BH): the weighted greedy strategy is still used to determine the lighting cells of each time slot, but we do not perform the interference clustering in the resource allocation stage. All 12 illuminated cells adopt a genetic algorithm to search the joint bandwidth and power assignment. The iteration times for each slot is set as 200, which is four times that in our proposed improved algorithm.

### 5.2. Performance of Our Proposed Algorithm

As mentioned above, LEO communication systems face the challenge of uneven space-time distribution of terrestrial service requirements, and an effective beam hopping strategy should be able to overcome this obstacle.

We first simulate in the scenarios of even and uneven traffic distribution in the cells. Specifically, in the scenario of even traffic distribution, the traffic intensity of each cell is similar, which is set as 400 packages. In the scenario of uneven traffic distribution, 20 hotspot cells among the 195 cells have a traffic intensity of 2100 packets, while the traffic intensity of the remaining non-hotspot cells is 210 packets. It is worth noting that, in the scenarios of even and uneven traffic distribution, the sum of the total traffic demand of all cells is roughly the same, but the traffic demand of hot spots in the uneven scenario are about 10 times that of non-hot spots. The comparison simulation results under the two scenarios are as follows.

**Figure 7:**
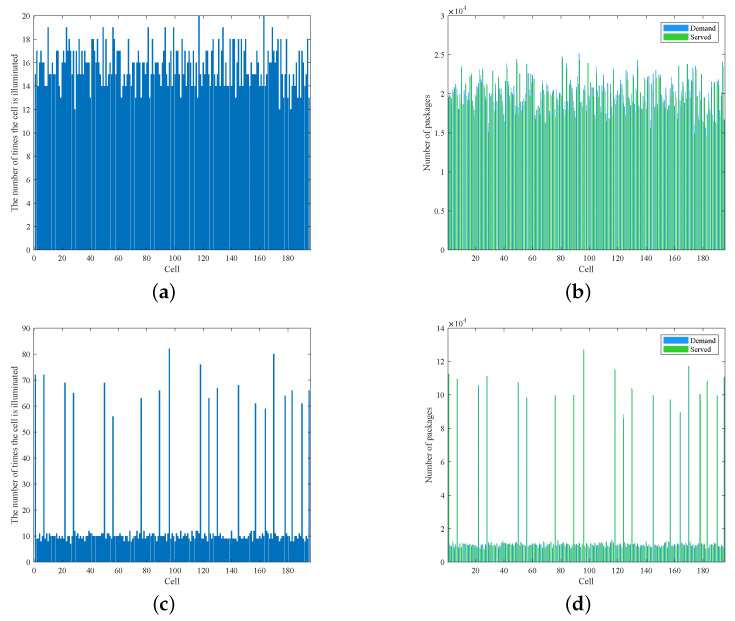
Comparison of even and uneven traffic distribution. (**a**) Illuminated times under even traffic distribution. (**b**) Demand response under even traffic distribution. (**c**) Illuminated times under uneven traffic distribution. (**d**) Demand response under uneven traffic distribution.

In the two scenarios of even and uneven distribution, the access success rates in the entire beam hopping period are 97.2% and 97.3%, respectively, and the average delays of all data packets are, respectively, 66.06 ms and 52.95 ms. As for the fairness between cells, the variance of the average delay of each cell is quite different in two scenarios, which comes to about 74 and 1188, respectively. The lighting times and demand response of each cell are shown in Figure 7.

As we can see from the picture, when the demand distribution is relatively even, the number of times that each cell is illuminated in the entire beam hopping period is also very uniform. When the demand distribution is obviously uneven, the beam resources are inclined to the hotspot cells, and the number of times of being illuminated increases significantly, thus leading to an obviously shorter transmission delay in them. Although the demand distribution of the two cases is quite different, under our algorithm, the demand of each cell has been responded to in time. That is to say, the beam hopping scheme we designed can provide on-demand services.

**Figure 8:**
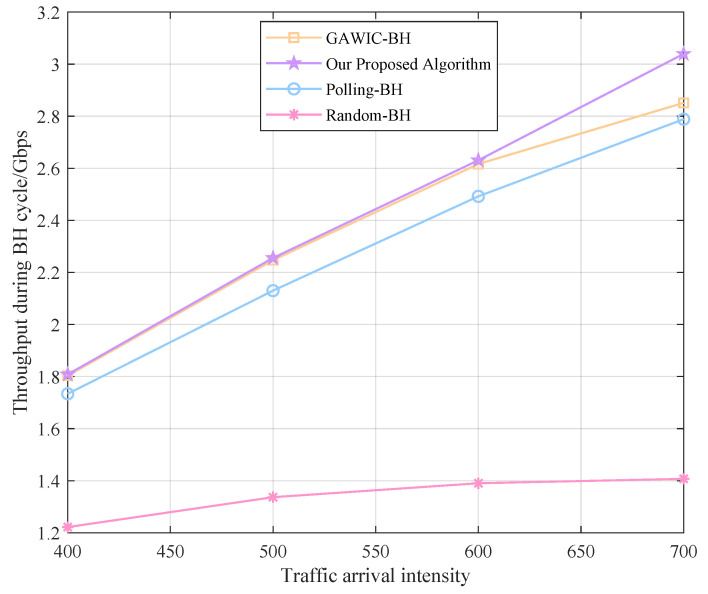
Throughput performance with different algorithms.

**Figure 9:**
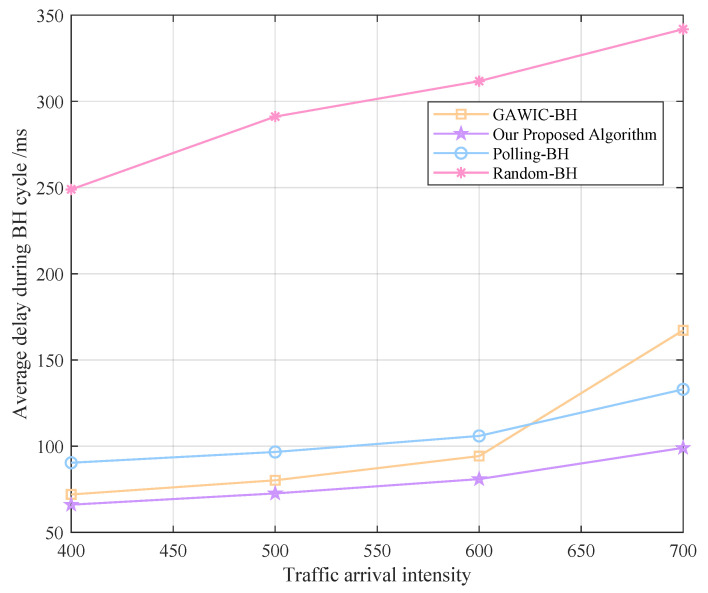
Average delay performance with different algorithms.

**Figure 10:**
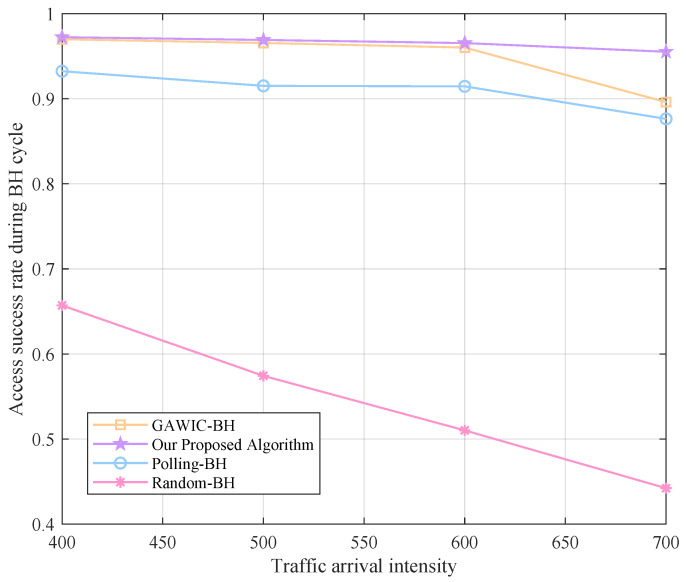
Access success rate performance with different algorithms.

**Figure 11:**
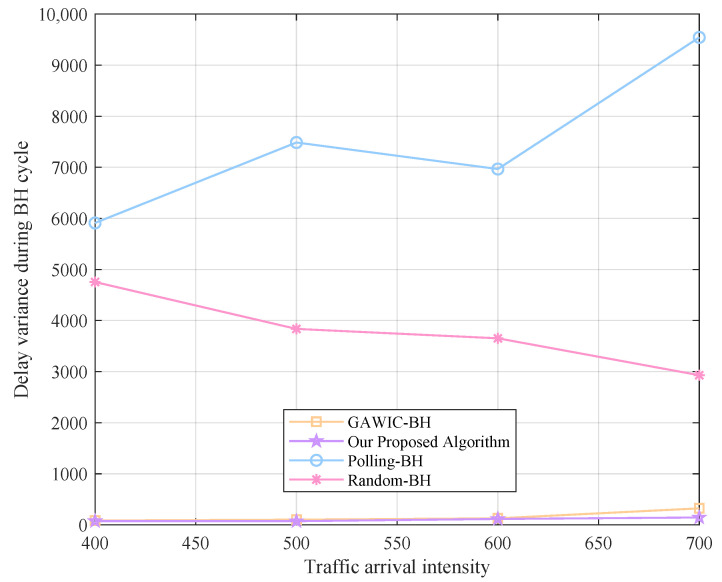
Fairness performance with different algorithms.

Next, we simulate under different traffic intensities to compare the performance of various algorithms. The traffic arrival intensity is set to 400, 500, 600 and 700 packages, respectively, and the system throughput, access success rate, average delay and fairness obtained by each algorithm are compared as shown in Figure 8, Figure 9, Figure 10 and Figure 11.

It is not difficult to observe from the figure that as the traffic intensity arriving at the cell continues to increase, the corresponding system throughput also increases gradually. Before the traffic intensity reaches 600, the growth is approximately linear. When the traffic demand further increases, the advantages of our proposed algorithm over the traditional genetic algorithm gradually emerge, and our algorithm can still maintain a stable throughput growth rate with a large traffic demand.

In terms of the average access delay of all data packets in the beam hopping period, among the four algorithms, our proposed algorithm obtains the lowest delay, with an average delay of 99.03 ms when the traffic intensity reaches 700 packets. As the traffic demand increases, the average delay obtained by the polling-BH algorithm is steadily about 33% higher than our algorithm, while the growth rate of the delay obtained by the GAWIC-BH algorithm is gradually increasing. When the demand is small, the delay obtained by the GAWIC-BH algorithm is about 9% higher than that of our algorithm, and when the intensity reaches 700, the delay obtained by the GAWIC-BH algorithm reaches 167 ms, which is about 68% higher than our algorithm.

As the traffic demand increases, the access success rate in the entire beam hopping period gradually decreases, but in general, the access success rate of the scheme using the genetic algorithm is higher than that of the polling scheme. This is because the order of serving cells in the polling scheme is relatively fixed, and it is impossible to schedule beams flexibly according to real-time demand, which will inevitably cause many data packets to be abandoned due to waiting for too long, and the access success rate will decrease accordingly. Our method performs better than GAWIC-BH, which is reflected in the fact that the access success rate decreases more smoothly. The access success rate of Random-BH is the lowest, and as the traffic intensity increases, the access success rate drops significantly.

The illumination pattern selection based on weighted greedy strategy ensures the service fairness of each cell. On the one hand, the weight of the amount allows the cells with more demand to be preferentially served, avoiding congestion caused by excessive demand that cannot be satisfied in time. On the other hand, the urgency weight is set, even if the demand in the cell is not high, and if it is not served for a long time, the urgency of the data packets in the cell will increase greatly, and the beam will be preferentially scheduled in the subsequent time to avoid access failure.

**Figure 12:**
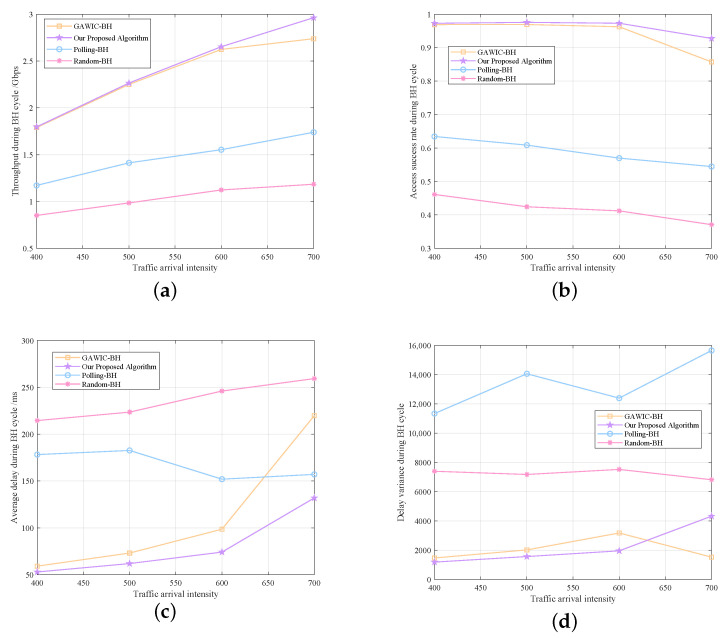
Comparison in uneven distribution scenario. (**a**) Throughput. (**b**) Access Success Rate. (**c**) Average Delay. (**d**) Delay Variance.

Under the scenario of uneven distribution of terrestrial traffic demand, several comparison simulations are carried out to show the superiority of our proposed algorithm. Twenty hotspot areas are set in 195 cells to simulate the performance of each algorithm for scheduling beam hopping resources under different traffic intensities. The traffic intensity is set as follows: the traffic demand in the hotspot cells is 10 times that of the non-hotspot cells. The sum of the traffic of all cells is the same as that in the even distribution scenario, corresponding to the traffic intensity from 400 to 700 packets in the even scenarios. The simulation results are shown in Figure 12, and the data comparison between the two scenarios is shown in Table 4 and Table 5.

As depicted in the above figures and tables, our proposed algorithm outperforms all the comparison algorithms on all the performance metrics. The performance curves of various algorithms are similar to those in the even distribution scenario. It is not difficult to observe that when the traffic distribution is uneven, our algorithm and GAWIC-BH can also achieve relatively high throughput and access success rate while the performance of Random-BH and Polling-BH deteriorated significantly due to inflexibility. Additionally, the average delay obtained by our mechanism and GAWIC-BH is even shorter than that in even distribution scenario when traffic demand is relatively small, indicating that the genetic algorithm is very suitable for adapting to uneven space-time distribution of demands. Meanwhile, the advantages of our proposed scheduling scheme in ensuring service fairness between cells are very prominent. When the traffic intensity increases, the advantages of our algorithm compared with GAWIC-BH gradually become obvious and it can achieve better performance with only 1/4 the number of iterations of GAWIC-BH.

**Table 4:** Comparison of the results of each algorithm in the two scenarios when traffic is small ^1^.

Beam Hopping Scheme	Traffic Distribution	Throughput (Gbps)	Access Success Rate	Average Delay (ms)	Delay Variance
Polling-BH	even	1.734	93.2%	90.38	5910
	uneven	**1.172**	**63.4%**	**178.25**	**11,336**
Random-BH	even	1.222	65.7%	248.94	4754
	uneven	**0.852**	**46.1%**	**214.55**	**7393**
GAWIC-BH	even	1.804	97.0%	71.99	85
	uneven	**1.790**	**96.9%**	**59.27**	**1471**
Proposed Algorithm	even	1.808	97.2%	66.06	74
	uneven	**1.797**	**97.3%**	**52.95**	**1188**

^1^ Small traffic demand is defined as: Poisson arrival intensity is 400 packages under even distribution, 2100 in hotspots and 210 in non-hotspots under uneven distribution.

**Table 5:** Comparison of the results of each algorithm in the two scenarios when traffic is large ^1^.

Beam Hopping Scheme	Traffic Distribution	Throughput (Gbps)	Access Success Rate	Average Delay (ms)	Delay Variance
Polling-BH	even	2.789	87.6%	132.96	9543
	uneven	**1.739**	**54.5%**	**157.10**	**15,643**
Random-BH	even	1.407	44.22%	341.91	2930
	uneven	**1.185**	**37.1%**	**259.35**	**6815**
GAWIC-BH	even	2.851	89.6%	167.20	324
	uneven	**2.738**	**85.7%**	**219.82**	**1527**
Our Algorithm	even	3.039	95.5%	99.03	145
	uneven	**2.962**	**92.7%**	**131.96**	**4326**

^1^ Large traffic demand is defined as: Poisson arrival intensity is 700 packages under even distribution, 3100 in hotspots and 310 in non-hotspots under uneven distribution.

## 6. Conclusions

In this paper, we proposed a beam-hopping resource scheduling algorithm to efficiently allocate time slots, bandwidth, and power resources in the LEO communication scenario that shares spectrum with GEO communication systems. While achieving interference avoidance, the throughput and access success rate of the system are improved, and the communication delay is reduced. First, the illumination pattern is determined through a weighted greedy strategy, which ensures the fairness of cell services to a certain extent. Then, the active cells in the current time slot are clustered by interference clustering to reduce the search space. Finally, the joint allocation of bandwidth and power is carried out in each cluster through a genetic algorithm with optimal control.

Simulation results show that compared with the polling or random beam hopping strategy, our algorithm can flexibly achieve on-demand services for differentiated user needs, and has more advantages in scenarios with uneven user demand distribution. Meanwhile, compared with the traditional genetic algorithm (GAWIC-BH), our improved algorithm can obtain a solution with better performance at 1/4 the number of iterations, and with the increase of traffic intensity, this advantage is gradually significant. Specifically, in the scenario of uneven traffic distribution, when the traffic intensity is large, the system capacity obtained by our proposed mechanism is 70.3%, 150%, and 8.2% higher than that of polling-BH, random BH and GAWIC-BH, respectively. Our mechanism achieves the maximum access success rate of 92.7% and minimum average delay of 132ms among all schemes while ensuring service fairness between cells.

With the development of satellite Internet, the efficient and flexible scheduling of low-orbit satellite resources is the focus of future research. This paper mainly focuses on downlink beam scheduling, and we can comprehensively take the uplink scenario into consideration in future research. Furthermore, considering the dynamic mobility of LEO satellites and the multiple coverage of mega-constellations, multi-satellite joint resource scheduling will be studied to achieve more flexible resource allocation and interference avoidance of GEO systems.

## Figures and Tables

**Table 1:** Antenna model simulation parameters.

Parameter	Notation	Value
LEO satellite orbit height	$H_{l}$	1050 km
LEO satellite beam radius	$R_{l}$	40 km
GEO terminal recieving antenna half power angle	$\theta_{3\mathrm{dB}}^{g,r}$	1.58${}^{\circ }$
LEO terminal recieving antenna half power angle	$\theta_{3\mathrm{dB}}^{l,r}$	2.06${}^{\circ }$
antenna efficiency	η	0.7
constant related to the field distribution	*N*	65

**Table 3:** System parameters.

Parameters	Value
Number of cells *N*	195
Number of hopping beams *K*	12
Frequency of downlink *f*	30 GHz
Total transmission power $P_{tot}$	360 W
Total available bandwidth $B_{tot}$	250 MHz
Number of sub-bands *m*	10
Interference threshold to GEO system $I_{th}$	−132.5 dBW
Packet size ps	1200 bit
Packet Poisson arrival rate ρ	2 $T_{slot}$
Number of time slots in a BH cycle *k*	256
Length of time slot $T_{slot}$	10 ms
Maximum queuing time $T_{ttl}$	50 $T_{slot}$
Weight of traffic amount $\omega_{1}$	0.5
Weight of traffic emergency $\omega_{2}$	0.5

## Data Availability

Not applicable.
